# Locke's View of the Hard Problem of Consciousness and Its Implications for Neuroscience and Computer Science

**DOI:** 10.3389/fpsyg.2017.01069

**Published:** 2017-06-23

**Authors:** John E. Lisman

**Affiliations:** Department of Biology, Brandeis UniversityWaltham, MA, United States

**Keywords:** qualia, NMDA-spike, CREB, sleepwalking, excitability, feedback

Modern neuroscience has come to accept the challenge of understanding consciousness (Crick and Koch, 2003; Dehaene and Changeux, 2011). A major finding has been the discovery that there are conscious and unconscious systems in the brain (top and bottom of Figure 1, Lisman and Sternberg, 2013) and the development of methods for manipulating whether a sensory input will be consciously or unconsciously processed (Lamme, 2006). Interestingly, even stimuli that are not consciously processed can stimulate emotions and actions (Flykt et al., 2007).

**Figure 1:**
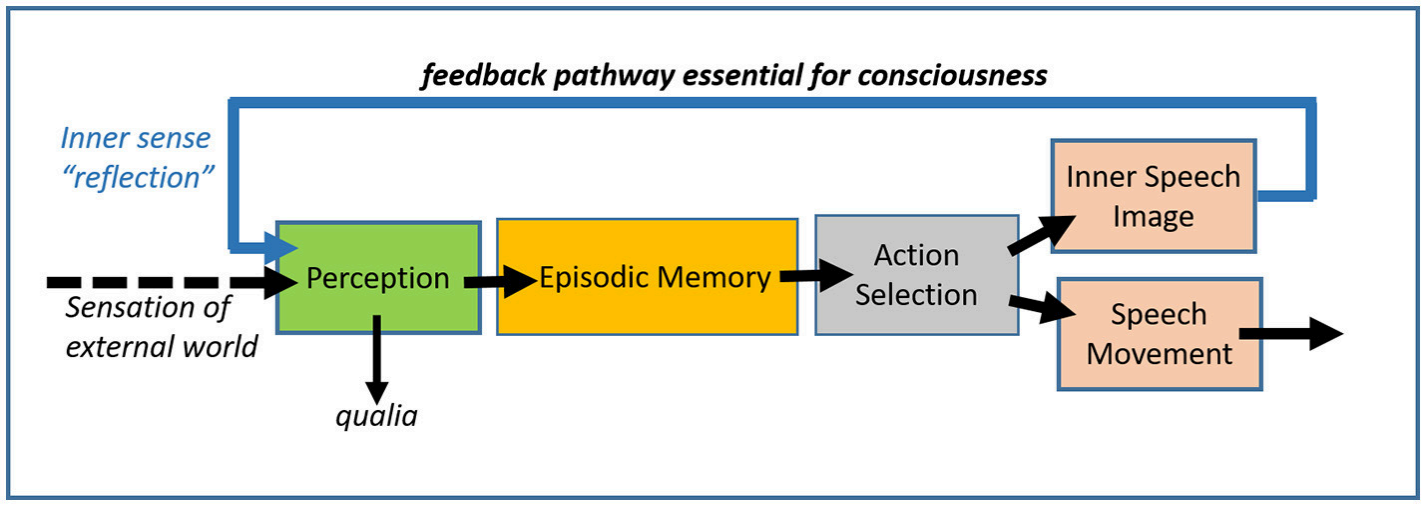
The unique neural pathway (blue feedback arrow) that, according to a neural interpretation of Locke, is essential for consciousness. The feedback pathway provides internal sensation (reflection) that, together with sensory input from the external world, provides the only inputs to the perceptual system. Inner speech and image generation are considered the result of the action selection mechanism. The action selection system utilizes information from perceptual and memory systems. There is also an unconscious system (not shown) that can perform stimulus/response functions.

Despite this progress, there is the strong sentiment that the “hard” problem of consciousness has not been addressed. The hard problem deals with the question of how the *feeling* of consciousness arises. We feel conscious and the word seems easy to relate to our experience, but defining it is difficult. This impedes elucidation of the neural underpinnings of consciousness and makes it impossible to take a clear stand on whether a computer could be conscious.

A recent meeting brought together experts in the hard problem, with two of the main protagonists being Daniel Dennett and David Chalmers. According to a report of this meeting (*New Yorker*, March 2017), Dennett believes that the brain is a collection of subsystems, with more advanced organisms having more subsystems. These subsystems interact to produce consciousness in a way that is greater than the sum of the parts. Moreover, consciousness is graded, with advanced organisms having much more of it than lower organisms. Chalmers' view is quite different. He believes that consciousness can only be understood by including processes outside of the brain. According to the article, he has been “gravitating toward ‘pan-proto-psychism’”—the idea that consciousness might be “a fundamental property of the universe upon which the brain somehow draws.”

I suggest that it is useful to consider a third idea about consciousness based on the ideas put forward by the philosopher John Locke (1603–1704) in his *Essay Concerning Human Understanding*, Book II, Chapter 1. Locke believed that there are two types of sensory input to the perceptual system, one from the sense organs and another from the “internal sense.” About this second input, Locke wrote:

“*This source of ideas every man has wholly in himself; and though it be not sense, as having nothing to do with external objects, yet it is very like it, and might properly enough be called internal sense. But as I call the other sensation, I call this reflection, the ideas it affords being such only as the mind gets by reflecting on its own operations within itself.”*

This framework then led Locke to coin the word *consciousness* and to define the action of the reflection process. Specifically, Locke's definition of consciousness is:

“*Perception of what passes in a man's own mind.”*

Locke's definition is interesting because it can be mapped onto brain processes. Thoughts take the form of inner speech or images. These result from the action selection/action production system that generates the *output* signals of the brain. Following Locke's idea, consciousness arises because these output signals are sent back to the sensory *input* regions of the brain that underlie perception. The specific neurobiological implication is that there is a major pathway that connects output structures of the brain to the perceptual apparatus of the brain, a pathway shown by the blue arrow in Figure 1. I use the word “major” here to emphasize that the feedback is not a simple analog signal that could be carried by a single axon but, rather, a substantial information flow that requires a multi-axon nerve pathway sufficient to carry information about words or images.

Acceptance of Locke's definition hinges on what one thinks would happen if the feedback pathway (blue arrow) was made non-functional. The upper system in Figure 1 could still process visual input and produce a motor output. What would be lacking is the perception of what passes in one's mind; there would be no reflection on what one saw. By Locke's definition, consciousness would not be present.

The above account deals with language, but Locke's definition can also be applied to other sensory modalities—for instance, visual images. If you had been bitten on the hand by a dog, the appearance of the dog might evoke from memory the image of your hand in the dog's jaw, but only if the feedback pathway was intact. Readers will have to decide whether they think that consciousness can survive elimination of the feedback pathway. In my view, it cannot.

Locke's definition bears on two other concepts, the stream of consciousness and qualia. A well-established concept in memory research is that a simple sensory cue can trigger the recall of a complex memory of which the cue is a part. The feedback pathway could provide such cues, thus giving rise to the next memory/thought, which would then serve as a further cue for the next memory/thought. In this way, a stream of consciousness could arise. Some people are troubled by racing thoughts that form an extremely strong stream of consciousness, as illustrated in the following example obtained from a website for the problem:

“*I always forget what I have to do. I'm so stupid. If I don't remember everything, I'll get fired. I don't know what I'll do if that happens. I should have taken that job I was offered six months ago. If I lose my job, I won't have any money. I need to work longer hours to keep this job. That just makes me more depressed. I'm so miserable. What am I going to do?”*

Locke's definition of consciousness leads to a surprising conclusion about the role of qualia in consciousness. The term “qualia” refers to the irreducible aspect of a sensation, e.g., red, and it is commonly thought that qualia are a defining property of consciousness. Note, however, that by Locke's definition, qualia, as produced by the perception system, could survive elimination of the feedback pathway. However, by Locke's definition, one would *not* be conscious. Thus, qualia may not be a marker of consciousness. Indeed, by a simple test, any computer capable of analysis of visual input would have qualia. In this test, one first shows a red stimulus to a computer vision system and types in “this is red.” Later, one shows the same red stimulus to the same computer system and asks whether the computer sees it. The answer would be “of course, it's just like what you showed me a while back.” On this analysis, qualia are due to the presence of information about the stimulus, as transformed by the stimulus-processing apparatus. It follows that standard computer architecture, in which information proceeds between processing blocks in a feedforward manner, is sufficient to generate qualia. However, such systems do not meet Locke's definition of consciousness because there is no feedback from output to input. Should a new computer architecture be developed in which output information was fed back to input processing systems, the computer might well be conscious.

## Author contributions

The author confirms being the sole contributor of this work and approved it for publication.

### Conflict of interest statement

The author declares that the research was conducted in the absence of any commercial or financial relationships that could be construed as a potential conflict of interest.
